# Cereal-Based Snack Bar with Added Plant Stanol Ester (Benecol®) Consumed between Meals Lowers Serum Total and LDL Cholesterol Effectively in Mildly to Moderately Hypercholesterolemic Subjects

**DOI:** 10.1155/2018/1463628

**Published:** 2018-05-02

**Authors:** Essi Sarkkinen, Mari Lyyra, Sakari Nieminen, Päivi Kuusisto, Ingmar Wester

**Affiliations:** ^1^Oy Medfiles Ltd., Volttikatu 5, P.O. Box 1627, 70701 Kuopio, Finland; ^2^Satucon/Itelasaretti, Koljonniemenkatu 2, 70100 Kuopio, Finland; ^3^Raisio Nutrition Ltd., P.O. Box 101, 21201 Raisio, Finland

## Abstract

The cholesterol-lowering effect of foods with added plant sterols or stanols consumed as snacks might be compromised. The purpose of this study was to confirm the cholesterol-lowering efficacy of a specially formulated cereal-based snack bar with added plant stanol ester (1.6 g plant stanols/day) when consumed between meals twice a day. In a double-blind, placebo-controlled, 4-week parallel-design study, 71 mildly to moderately hypercholesterolemic subjects were randomized into one of two groups, stanol or placebo group. Subjects were advised to replace their ordinary snacks with test products in an isocaloric manner and otherwise keep their habitual diet unchanged. The study showed that a snack bar product with added plant stanol ester lowered LDL and non-HDL cholesterol by 8.6% and 9.2% (mean%-change), respectively, as compared to the placebo product. The change in LDL cholesterol was statistically significantly different (*P* = 0.001) between the groups while the change in HDL cholesterol or triglycerides did not differ between the groups. In conclusion, the cereal-based snack bar with added plant stanol ester ingested without a meal reduced LDL cholesterol significantly without affecting HDL cholesterol or triglyceride concentrations in mildly hypercholesterolemic men and women. The study is registered as NCT03284918.

## 1. Introduction

The cholesterol-lowering properties of plant sterols have been known since the 1950s [1–3]. Plant sterols decrease the absorption of both biliary and dietary cholesterol from the small intestine. Plant stanols are the saturated form of plant sterols. Both plant sterols and stanols reduce cholesterol absorption and lead to a reduction in LDL cholesterol when consumed in high enough daily amounts. Plant sterols and stanols result in similar LDL lowering with a daily intake in the range of 2 g/day [4, 5].

Plant sterols and stanols show low absorption rates [6]. Plant stanols are absorbed less efficiently and the absorbed plant stanols are eliminated faster from the body than the corresponding plant sterols [6]. Plant stanols also reduce the absorption of plant sterols, leading to reduced plasma plant sterol concentrations, whereas plant sterol consumption may lead to an increase in the plasma plant sterol concentrations [6]. The possible atherogenicity of circulating plant sterols, but not of plant stanols, has been the topic of a scientific debate during the past years as various studies and meta-analyses have produced conflicting result [7]. The scientific evaluation is hampered by the lack of method standardization for analysing plasma or serum plant sterols and stanols [8]. The European Atherosclerosis Society Consensus Panel on Phytosterols [7] concluded in 2014 that the data available does not provide a scientific basis to discourage the use of plant sterols or plant stanols containing functional foods in this respect.

Fat-soluble plant stanol ester was developed to be used as an ingredient in food products to achieve significant, clinically relevant reductions in serum total and LDL cholesterol levels. According to the European Food Safety Authority's evaluation [9], a daily intake of 1.5–2.4 grams of plant stanols lowers the serum LDL cholesterol by 7–10% and a daily intake of 2.5–3.0 grams of plant stanols lowers the serum LDL cholesterol by 10–12.5% when used in yellow fat spreads, dairy products, mayonnaise, and salad dressings. The cholesterol-lowering efficacy of plant stanol ester has been confirmed in more than 80 clinical studies. In most of them, the efficacy of plant stanol ester has been studied when incorporated into mayonnaise, regular or low-fat spreads, or yoghurts [3, 5, 9–11].

The expected efficacy of plant stanol ester has been demonstrated in several studies in which food products with added plant stanol ester are consumed with a meal [10–14]. When consumed as part of a meal, plant stanol ester is effectively hydrolysed to plant stanols which reduce serum cholesterol by replacing dietary (external) and biliary (“internal”) cholesterol in the mixed micelles during gastrointestinal handling of food [15]. Subsequently, less cholesterol is available for absorption into the enterocytes. It is likely that there are also other thus far unknown mechanisms of action of plant stanols. One indication of such a mechanism is the similar cholesterol-lowering efficacy of plant stanol ester regardless of whether the daily dose is consumed in one or several portions as part of a meal [16, 17]. If the only mechanism of action was the micellar effect then one would expect that it would be necessary to consume plant stanol ester with all daily meals for an optimal cholesterol-lowering effect.

The current recommended mode of consumption of food products with added plant stanol ester is with a meal [17–19]. The most common food product applications with added plant stanol ester are margarine-type spreads and dessert-type yoghurt drinks. These are naturally consumed with or in conjunction with a meal. The current trend of food habits indicates increased snacking-type eating [20]. Thus, there is interest in snack-type food product applications with added plant stanol ester.

There is some previous evidence about compromised cholesterol-lowering efficacy when yoghurt drinks with added plant sterol esters are consumed on an empty stomach [21, 22]. Doornbos et al. [21] found that the consumption of 3 g plant sterols in a 100 ml yoghurt drink on an empty stomach 30 minutes before breakfast lowered the serum LDL cholesterol significantly less as compared to the consumption of the same product as part of a meal. Keszthelyi et al. [22] showed that consumption of a plant sterol ester yoghurt drink 45 minutes prior to consuming a meal led to a fast gastric emptying and did not sufficiently trigger gallbladder contraction.

Kriengsinyos et al. [18] found that a plant stanol ester oat biscuit delivering 2 g of plant stanols did not reduce LDL cholesterol when consumed as such without any other foods, whereas the expected LDL lowering was obtained when the plant stanol ester oat biscuit was consumed with other food. Kriengsinyos et al. [18] speculated that the fat content of the biscuit may not have been high enough to sufficiently trigger gallbladder contraction when the biscuit was consumed without other food.

The cholesterol-lowering efficacy of snack-type products may thus depend on the food matrices to which plant stanol ester or plant sterol ester is added. The effect of the food matrix is apparently not so crucial when foods with added plant stanols or sterols are ingested with a meal [19, 22, 23]. Liquid foods such as drinkable yoghurts are expected to have quite a fast gastric transit time, especially when consumed on an empty stomach, whereas the digestion of solid products includes additional “processing steps” such as chewing with release of lingual lipase and subsequent partial hydrolyses of triglyceride fat in the stomach. For solid plant stanol ester foods, good efficacy further requires effective release of the plant stanol ester and fat from the food matrix in stomach and effective emulsification of the released fat-plant stanol ester blend. These aspects must be considered in the formulation of foods with added plant stanol ester that are typically consumed as snacks. It is therefore important to confirm the cholesterol-lowering efficacy of new plant stanol ester products mainly consumed as snacks by a clinical study. The aim of the present study was to test the lipid-lowering efficacy of a specially formulated cereal-based snack bar with added plant stanol ester when consumed in-between meals.

## 2. Materials and Methods

### 2.1. Study Subjects

A sample size of 35 randomized subjects per study group (total *n* = 70) was required in order to detect the assumed difference of 7.5 percentage units in mean percentage change of LDL cholesterol with a probability of 80% at *α* level of 0.05 (allowing a 15% drop-out rate per protocol population).

A total of 94 subjects were recruited mainly through advertising (including newspapers, Internet, and public notice boards) for the study from the Northern Savo area, Finland. The subjects were prescreened over the telephone. After the prescreening, the eligible 94 volunteers were screened and 23 did not meet the inclusion criteria and were excluded. The remaining 71 subjects were randomized. One subject in the stanol group discontinued the study due to gastrointestinal symptoms before Visit 4. In all, 70 subjects completed the study.

To be eligible for inclusion, the subject had to fulfil all of the following criteria: age 18–70 years; serum total cholesterol 5.2–8.5 mmol/l; and serum triglycerides < 3 mmol/l. The presence of any of the following criteria excluded the subject from participating in the study: abnormal liver, kidney, and thyroid function; use of lipid-lowering medication; consumption of other plant sterol or plant stanol containing food products or supplements or other foods or supplements for cholesterol-lowering one month prior to the baseline visit; history of unstable coronary artery disease within the previous 6 months; diagnosed type 1 or type 2 diabetes requiring medical treatment; history of recent temporal ischemic attack or malignant diseases (<5 yrs); celiac disease, or allergy or intolerance to the ingredients of the test products; medically prescribed diet or a special diet (such as a very low-calorie diet, vegan, or gluten-free diet) or medication for slimming (such as an obesity drug); subjects with an alcohol abuse problem; pregnancy or planned pregnancy or breastfeeding. A signed written informed consent was required from all subjects.

### 2.2. Study Design

This study was a randomized, double-blind, placebo-controlled 4-week intervention study following a two-arm parallel design. After the screening phase, all subjects were randomized into one of the two study groups: Active (stanol) or placebo. Centralized nonconcealed allocation was used in the study. Stratified randomization according to sex was used. Randomized subjects (*n* = 71) were advised to replace their ordinary snacks with the test products (either with the active or with placebo snack bar) for 4 weeks. The study was carried out under double-blind conditions. At screening, baseline, and 4-week visit, 10–12-hour fasting blood samples were drawn to screen health status and to measure serum lipids.

The study was conducted following the ethical principles of the Declaration of Helsinki and Good Clinical Practice as applicable for dietary interventions. The study was approved by an IEC, Research Ethics Committee of the Hospital District of Northern Savo, Kuopio, Finland.

### 2.3. Test Products and Background Diet

The stanol group consumed a specially formulated cereal-based bar with added plant stanol ester (Raisio Nutrition Ltd., Raisio, Finland) as a snack between meals twice a day one in the morning and one in the afternoon as part of their habitual diet. The plant stanol ester bar was formulated in such a way that effective release of the plant stanol ester and fat from the bars was ensured in the stomach. The planned portion provided 2 times 0.8 g plant stanols, that is, altogether 1.6 g plant stanols daily as plant stanol ester. A similar cereal-based bar without added plant stanol ester and used in a similar manner as the active product was consumed by the subjects in the placebo group. The study products were packed in nontransparent white foils with three-digit code labels. Subjects were counselled by a nutritionist to replace their ordinary snack products (like snack bars, bread, biscuits, cereals, etc.) with the test products in an isocaloric manner, and otherwise to keep their habitual diet, medication, other lifestyles (e.g., smoking and exercise), and body weight constant during the study. Bars were instructed to be used between the main meals, and the subjects recorded both the timing for the bar ingestion and the timing for preceding meals in to a diary. Use of other products or foods or food supplements intended for cholesterol lowering was not allowed during the intervention.

The nutrient composition of the test products is provided in Table 1. The oat-based, partly milk chocolate-coated test bars provided 272–278 kcal (570–585 KJ) energy, 10.4–11.2 g fat, 19.6–20 g sugars, and 12.2–12.4 g dietary fibres daily. The plant stanol ester bar delivered 1.54 g plant stanols and 0.1 g plant sterols per day. The sterol composition was as follows: sitostanol 85.6%, campestanol 8.0%, sitosterol 3.0%, and campesterol 2.5%. The subjects were guided to consume the test bars as in-between meal snacks without any other foods and with noncaloric drinks only.

The subjects recorded the use of the test product and possible changes in their health and life style parameters daily on a diary.

The compliance of the test product use was measured based on visit scheduling (number of days and bars that should have been consumed) and recordings (number of bars consumed) in the diary. In addition, a check was made against the product delivery logs (matching of the delivered and returned products to diary-recorded amounts). The criteria for compliance were set as follows: a subject taking less than 80% or more than 120% of the planned intake was considered noncompliant and planned to be excluded from the per protocol analysis. However, no such withdrawal took place in the study. In addition, subjects recorded the timing for ingestion of the bars (morning and afternoon) and the time to the previous meal or snack in the diary.

A three-day food record (including one day off work) was used to monitor the background diet before and during the intervention. The subjects were asked to record the types of food/drinks and sizes of meals and snacks with the help of a portion-size picture booklet, and a nutritionist checked the completeness of the recordings during the visits to the clinical site. Based on the food records, the nutrient content excluding the test products was calculated by using Aivo-Diet nutrient calculation programme utilizing national Fineli (Finnish Food Composition) database. Key energy nutrients (total fat, saturated fat, monounsaturated fat, polyunsaturated fat, protein, and carbohydrate) were calculated as grams and as energy proportions, and cholesterol and fibre were calculated as mg/day and g/day, respectively. Subjects recorded the use of test products and possible food supplements to the study diary but they were not counted in the nutrient calculation.

### 2.4. Anthropometric Measurements

A structured interview on previous and current diseases, current medication, alcohol and tobacco consumption, and use of dietary supplements was carried out at the screening visit by the study personnel.

All anthropometric measurements were performed by a trained study nurse. The body height was measured without shoes in light clothing. The height was measured with the subjects standing straight, hands beside the body, shoulders relaxed, and heels together. The head of the subject had to be in the so-called Frankfort position, auditory canal being horizontal on the same level as the top of the lower eyelids. The result was recorded to the nearest crossed half a centimetre (0.5 cm).

Body weight was measured with a calibrated digital scale. The subjects were weighed after an overnight fast (10–12 h), while they were wearing light indoor clothing and no shoes. If the difference of two measurements was bigger than 0.5 kg, the measurement was repeated. The mean of the two measurements was recorded in the CRF (Case Report Form). Weight was recorded to the nearest 0.1 kg. Body mass index (BMI) was calculated with the following formula: body mass index (kg/m^2^) = body mass (kg)/[height (m)]^2^.

### 2.5. Laboratory and Safety Assessments

All blood samples were collected after a 10–12 h overnight fasting. The subjects were reminded about the fasting with a SMS message. They were allowed to drink a glass of water at home on the blood test mornings. If water was ingested at the first test occasion, this was encouraged to be repeated also on the following test mornings. Routine safety parameters (B-Hemoglobin, B-Haematocrite, B-Erythrocytes, E-MCV, B-Tromb, B-Leucocytes, and S-Glutamyl transferase) were measured at the beginning and at the end of the study. The study site drew, prehandled, and centrifuged the blood samples according to laboratory's (Medix) instructions. The blood samples were analysed with standardized clinical chemistry and hematology methods at Yhtyneet Medix Laboratory, Helsinki, Finland. Plasma total cholesterol, lipoprotein cholesterol (LDL and HDL), and triglycerides were analysed by enzymatic methods. HDL cholesterol and LDL cholesterol were analysed by direct methods using automatic analyser (ADVIA 1800 System, Siemens Healthcare Inc. Tarrytown, NY USA). Commercial reagents were used: Concentrated Cholesterol Reagent (CHOL_c) Ref. 04993681, Direct HDL Cholesterol (D-HDL) Ref. 07511947, LDL Cholesterol Direct (LDL) Ref: 09796248, and Triglycerides_2 (Trig_2) Ref. 10335892, Siemens Healthcare Diagnostics Inc., USA. In addition, serum LDL cholesterol was also calculated with the Friedewald formula as follows: fS-Kol-LDL = fS-Kol − fS-Kol-HDL − (fS-Trigly/2.2) as a secondary analysis [24]. Serum non-HDL cholesterol was calculated as a difference of total cholesterol and HDL cholesterol with the following formula: non-HDL cholesterol = (fS-tot-Kol) − (fS-HDL-Kol).

Adverse events and all other symptoms observed by the investigator or spontaneously reported by the study subjects into the diary were systematically recorded.

### 2.6. Statistical Analyses

The study was an intervention study with two parallel groups (Active (stanol) and placebo) evaluating the differences between the two groups as regards the lipid-lowering efficacy. The primary outcome variable was the percentage change in LDL cholesterol during the 4 wk intervention (mean Δ  4 wk − 0 wk/0 wk*∗*100) between the groups. The primary outcome was evaluated using an ANOVA model with the treatment (PRODUCT) as a main effect (equal to *t*-test). When analysing differences in absolute values, the analysis of covariance (ANCOVA) model was used to adjust for the possible differences in baseline (0 weeks). The magnitude of the treatment differences was evaluated by constructing 95% confidence intervals of the differences.

If the assumptions of the ANCOVA model failed to hold on the original scale of percentual/absolute changes, the most common transformations (i.e., square or logarithmic) or a nonparametric test were considered. *P* values less than 0.05 (2-sided) were regarded as statistically significant.

The secondary variables (pretrial characteristics, serum total, and lipoprotein lipids other than the LDL cholesterol) were reported using descriptive statistics and analysed using methods similar to the primary parameter. The safety analysis included all subjects that received the investigational products (including placebo). One subject in the stanol group discontinued the study. No imputations were done. The study had one primary parameter with predefined analysis method. Thus, no multiplicity corrections were required.

## 3. Results

### 3.1. Subject Characteristics and Compliance

Pretrial characteristics of the subjects who completed the study are presented in Table 2. Subjects were middle-aged mildly hypercholesterolemic men and women. Subjects' mean LDL cholesterol was 4.2 mmol/l at screening and reduced to some extent already before randomization to 4.1 mmol/l. Subjects were slightly obese, and the mean weight remained stable in both groups during the intervention (Table 2). There were no major differences in any baseline laboratory measurements between the study groups (Tables 2 and 4) or safety parameters (data not shown).

The subjects were not allowed to have any lipid-lowering medication or major chronic diseases. Most commonly reported conditions in their medical history were musculoskeletal diseases and circulatory diseases in both groups (27.1% and 25.7%, resp., in total population). Medication related to the cardiovascular system, such as medication to lower blood pressure, was the most common ongoing medication. The most frequent intermittent concomitant medication during the intervention was the use of analgesics. A major part of the subjects (79%) did use some allowed food supplements. Fat-soluble vitamins and combination of vitamin and minerals were the most common products used. Use of food supplements remained stable during the study.

Test product compliance was very good since there were only few occasional minor deviations (from 1 to a maximum of 4 bars during the whole intervention period) from the instructed use (two test snack bars daily consumed on separate occasions). Thus, the overall compliance rate was 98.8% in both the placebo group and the stanol group. In addition, subjects were instructed to use the snack bars between the meals. The median time from the previous meal was 2.5 hours (0.8,22; min, max) in the placebo group and 2.3 hours in the stanol group (0.4, 20; min, max). The maximum time of 20–22 hours from the previous meals was due to a couple of night shift workers. The actual daily intake of plant stanols plus sterols was, according to the analysis of the test products, 1.65 g/d in the stanol group and 0.07 g/d in the placebo group (Table 1).

### 3.2. Background Diet and Lifestyle

Energy and nutrient intakes are presented without the nutrients obtained from the test snack bars (Table 3) to more easily figure out potential changes in the background diet. Detailed nutritional compositions of the test products are presented in Table 1. According to the three-day food recording, the background diet of the subjects remained otherwise stable except the assumed reduction in energy intake and energy nutrients, mainly protein and carbohydrates, due to removing the ordinary snacks from the diets. Based on the food records, the reduction of energy intake was about 580 kJ in the placebo group and 720 kJ in the stanol group. The cereal-based snack bars provided 1140–1170 kJ daily altogether, so the targeted isocaloric replacement was not fully accomplished in either of the two groups based on the food records. However, the increase in energy intake did not lead to any weight gain during the intervention (Table 2). No other major compositional changes took place in the background diet (Table 3). Most importantly, no major differences in the background diet were detected between the study groups during the intervention period or the run-in period (Table 3).

None of the subjects were regular smokers and only 3 in the placebo group and 6 in the stanol group used alcohol according to the lifestyle interview, and only minor changes in lifestyle took place during the intervention.

### 3.3. Plasma Lipid Variables

**Figure 1:**
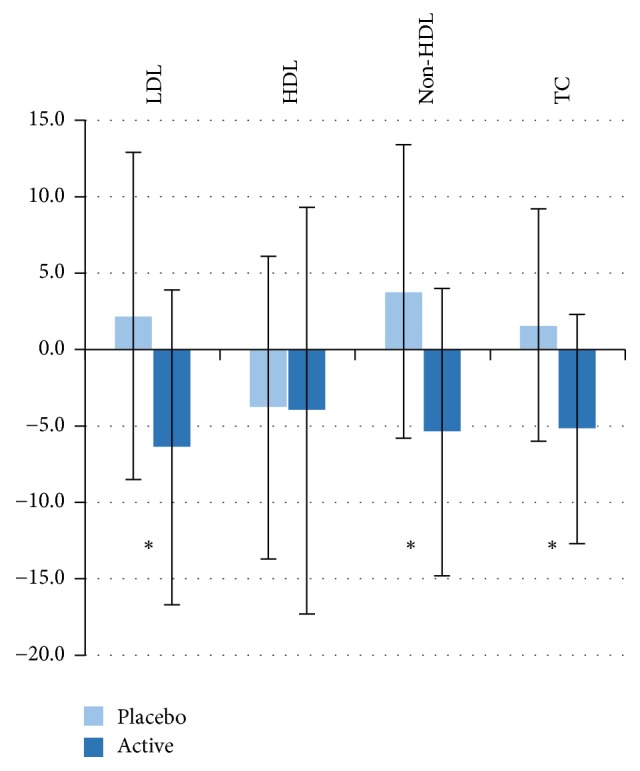
Percentage changes in serum total (TC) and lipoprotein lipids (LDL cholesterol, HDL cholesterol, and non-HDL cholesterol) during 4-week intervention in Placebo and Stanol group (mean ± SD). ^*∗*^Statistically significant difference between the groups *P* ≤ 0.001.

During the 4-week intervention, the use of the cereal-based bar with added plant stanol ester as an in-between meal snack twice a day reduced LDL cholesterol by 6.4%, while LDL cholesterol increased in the placebo group by 2.2%. The net difference in the primary outcome (percentage change of LDL cholesterol 0 versus 4 weeks) in comparison to placebo was thus 8.6% (*P* = 0.001, ANOVA model, Table 4, Figure 1). The net difference in serum LDL cholesterol calculated with the Friedewald formula between the stanol and placebo groups was somewhat larger, 9.6%. The respective net difference in total cholesterol change was slightly less, −6.8%, in reference to placebo, but this was also statistically significant (*P* < 0.001). Consequently, the mean value of both total and LDL cholesterol was significantly lower (*P* < 0.01, Table 4) in the stanol group at the end of intervention compared to placebo. The net difference in non-HDL percentage during the 4-week intervention was −9.2% between the Placebo and stanol group (*P* < 0.001, ANOVA model, Table 4). There were no major differences between the groups in lipid variables at baseline, and thus the secondary analysis for absolute LDL or total cholesterol change (0 versus 4 weeks) using baseline value as covariate provided similar result as the primary comparison.

HDL cholesterol decreased only slightly (*P* < 0.05) in both groups, and thus the difference between the groups was not significant neither for the change variables nor for the mean values at end of intervention. Serum triglyceride increased significantly (*P* < 0.05) in the placebo group, but remained stable in the stanol group. However, due to the large variation in the difference between the groups, triglyceride variables were not statistically significant.

### 3.4. Safety Parameters

The safety laboratory parameters (B-Hemoglobin, B-Haematocrite, B-Erythrocytes, E-MCV, B-Tromb, B-Leucocytes, and S-Glutamyl transferase) remained stable and within the reference values in both groups during the intervention (data not shown). The most frequently reported adverse effects were mild or moderate gastrointestinal (GI) symptoms (32% versus 29% of the subjects reported; stanol versus placebo) and respiratory symptoms (32% versus 23%). About 30% in the stanol group estimated that GI symptoms were possibly, probably, or definitely related to the test products, while respective figure in the placebo group was slightly less, 24%. The GI symptoms were in most cases mild gastrointestinal discomfort which is not likely to be attributed to the plant stanol ester, but rather to other constituents of the food matrix, such as the fibre contained in the test bars. Respiratory symptoms were in most cases ordinary flu and in all cases rated “not to be related” or “unlikely related” to the use of test products. No serious adverse events occurred during the study.

## 4. Discussion

The present study showed in mildly to moderately hypercholesterolemic subjects that a specially formulated cereal-based snack bar with added plant stanol ester (1.6 g plant stanols/d) lowers LDL and non-HDL cholesterol significantly and efficiently, about 9%, in reference to the placebo product. Importantly, this reduction took place despite the snack bars being ingested between meals so that the mean time to the previous meal was 2.5 hours in the stanol group.

The observed reduction in LDL cholesterol in the present study was comparable with or greater than observed in previous studies using solid cereal-based food matrices [23, 25–27]. Clifton and coworkers [25] expressed the view that the LDL-lowering efficacy of plant sterol/stanol products varies between different food matrices. In solid cereal-based matrices the LDL-lowering efficacy has been smaller than in milk- or fat-based matrices [23, 25–27]. The foods tested by Clifton and coworkers [25] were most probably consumed as part of meals.

In the study by Polagruto et al. [23] the placebo-controlled reduction in LDL cholesterol remained at 6% although the daily plant sterol intake from the test bar was slightly higher (1.8/day) and the test bar was ingested not more than 30 minutes apart from the meal. A similar LDL-lowering effect (−5.4%) was obtained in a tall oil sterol group in a study comparing the cholesterol-lowering efficacy of plant sterols (1.8 g/d), glucomannan, and combination of these two in snack bars [26] consumed between meals. In a study with rye bread enriched with nonesterified plant sterol [27], a daily intake of 2 g plant sterols reduced LDL cholesterol by 8.1% versus control, while doubling the plant sterol intake to 4 g/day resulted in a 10.4% LDL lowering versus control. In the Söderholm et al. study [27], the test breads were ingested at least partly together with meals.

Kriengsinyos et al. [18] studied the lipid-lowering effect of a daily consumed biscuit containing 2 g of plant stanols delivered as plant stanol esters in a double-blind, placebo-controlled parallel-design study in mildly to moderately hypercholesterolemic volunteers. The study subjects first consumed a control biscuit once a day for a two-week run-in period. The subjects were then randomized either to the plant stanol ester biscuit or to the control biscuit group for an intervention period of four weeks. The subjects were free to consume the daily biscuit as they wished. The mean serum total cholesterol (TC) and LDL cholesterol were reduced by 4.9% and 6.1%, respectively, compared to control. Interestingly, a significantly higher reduction in LDL cholesterol (8.9%) was measured in those subject that consumed the plant stanol ester biscuit with a meal compared to only a 0.9% reduction in LDL cholesterol in subjects consuming the plant stanol ester biscuit as such without other food. The test biscuits delivered approximately 5.0 grams of fat with some protein and carbohydrate besides providing 120 kcal (502 KJ) per biscuit. Kriengsinyos et al. [18] speculated that the fat content of the biscuit may not have been high enough to sufficiently trigger gallbladder contraction.

The mechanism(s) of action by which plant sterols and stanols reduce cholesterol absorption are not yet fully known. The main mechanism of action is considered to be that plant stanols reduce serum cholesterol by replacing dietary (external) and biliary (“internal”) cholesterol in the mixed micelles during gastrointestinal handling of food [28]. In addition to competing for space in the mixed micelles, plant stanols are considered to interfere in the cellular cholesterol metabolism of the enterocyte by inhibiting chylomicron formation by making cholesterol less available in the intestinal cells and competing with cholesterol transporters [15, 28, 29].

In order for plant stanol ester to effectively reduce cholesterol absorption, it needs to be hydrolysed by the pancreatic cholesterol esterase enzyme to free plant stanols and fatty acids in the upper part of the small intestine. A prerequisite for this hydrolysis to take place is that the snack food product is formulated so that the plant stanol ester and some of the triglyceride fat is effectively released from the food matrix in the stomach. The snack food composition must be such that its component/components will induce gall bladder contraction and release of pancreatic lipases. The cereal bar with added plant stanol ester was formulated according to these principles and the obtained LDL-lowering results proved that this concept is working.

In this study there were no major differences in the baseline characteristics of the subjects between the groups. Compliance to the test product use was exceptionally high in the present study. Furthermore, according to the food records, there were no major differences in the background diets between the study groups neither at baseline nor during the intervention. Based on the food records, the targeted isocaloric replacement of the other snacks with the test bars was not fully accomplished since the energy reduction in the background diet was not fully in accordance with the daily energy derived from test bars, but about 500 kJ (120 kcal) less. This strongly indicates that it was not possible to reduce the energy intake from other snacks so that it would equal to the energy content of the two bars. A long-term increase of daily energy of 500 kJ (120 kcal) is expected to lead to an increase in body weight. However, in the present study body weight remained unchanged. Therefore, commercial applications of a cereal bar with added plant stanol ester should rather be based on the concept of one cereal bar per day. This concept is worthwhile to be tested in future studies. Previous studies have shown that the daily intake of plant stanols can be consumed in one serving without compromising the LDL-lowering effect [16, 17]. Since no significant changes were seen in the body weight within or between groups (Table 2) we concluded that seen reduction in the energy intake did not contribute to the lipid lowering observed in the stanol group. Altogether the obtained LDL cholesterol-lowering effect in the current study can be ascribed to the plant stanol ester in the cereal-based snack bar.

The present study demonstrated that also cereal-based snack bar products with added plant stanol ester can be a feasible and safe vehicle or food application for effective LDL lowering in a hypocholesterolemic diet. On the other hand, it emphasizes the importance to test each food application and food matrix separately to verify the cholesterol-lowering efficacy of each application. This is because an optimal cholesterol-lowering efficacy of plant stanols and sterols seems to be dependent both on the food matrix and/or its constituents, and whether the food is consumed with a meal or without a meal. This is of particular importance for snack products since they are typically ingested without other foods. One efficacy-limiting step for plant stanols or sterols incorporated into solid foods is the potentially reduced or slow release of the fat and added plant sterols/stanols from the matrix in the stomach. A prerequisite for the good cholesterol-lowering efficacy is an effective release of the plant stanol ester and fat from the food matrix and an effective emulsification with the fat phase in the stomach. In the present study, the cereal snack bars were specifically formulated to facilitate fast release of the plant stanol ester and fat of the test bar in the stomach.

## 5. Conclusion

In conclusion, a specially formulated cereal-based snack bar product with added plant stanol ester ingested in-between meals twice a day reduced LDL cholesterol significantly without affecting HDL cholesterol or triglyceride concentrations in mildly to moderately hypercholesterolemic men and women. The product concept and food matrix used provide a feasible, effective, and safe alternative to incorporate plant stanol ester into a cholesterol-lowering diet.

## Figures and Tables

**Table 1:** Nutrient composition of the placebo and plant stanol ester enriched (stanol) test bars per 100 g and per one bar (35 g).

Nutrients	Placebo bar		Stanol bar	
	per 100 g	per 35 g	per 100 g	per 35 g
Energy (kcal)	399	139	390	136
Energy (kJ)	1670	585	1630	570
Fat	16	5.6	14.8	5.2
of which SAFA	6.5	1.8	4.8	1.7
Carbohydrates (g/d)	51	18	51	18
of which sugars (g)	28	10	28	9.8
Dietary fiber (g)	17.6	6.1	17.6	6.2
Protein (g)	5.5	1.9	5.5	1.9
Salt (g)	0.5	0.18	0.5	0.18
Plant sterols (g)	0,07	0,02	0,15	0,05
Plant stanols (g)	0.03	0.01	2.21	0.77

**Table 2:** Pretrial characteristics^1^ of the subjects in placebo and stanol groups.

	Placebo group (*N* = 34)	Stanol group (*N* = 36)
Gender (male /female) (*n*)	12/22	14/22
Ethnicity		
Caucasian (*n*)	34	36
Age (years)	58.3 ± 7.9	59.1 ± 7.7
Weight (kg) at		
Screening	74.4 ± 14.9	75.9 ± 15.8
Baseline	74.5 ± 15.0	75.8 ± 15.6
4 weeks	74.5 ± 15.0	76.0 ± 15.8
Height (cm) at Screening	166.7 ± 9.6	168.5 ± 9.3
Body mass index (kg/m^2^)		
Screening	26.7 ± 4.5	26.6 ± 4.9
Baseline	26.8 ± 4.6	26.6 ± 4.8
4 weeks	26.7 ± 4.5	26.7 ± 4.9
Serum glucose (mmol/l)	5.0 ± 0.4	5.2 ± 0.5
S-Thyroid stimulating hormone (mU/l)	2.2 ± 1.2	2.1 ± 1.1
S-Creatinine (*μ*mol/l)	67.2 ± 14.5	71.5 ± 12.8

^1^Values are means ± SD for continuous variables. There were no statistically significant differences in the baseline characteristics between the groups.

**Table 3:** Energy and nutrient intakes^1^ from background diet during the run-in and intervention in the study groups.

Nutrients	Placebo group		Stanol group	
	Run-in	Intervention^2^	Run-in	Intervention^2^
Energy (KJ)	7604 ± 1518	7021 ± 1679	7669 ± 2002	6949 ± 2217
Carbohydrates (g/d)	198 ± 50	180 ± 53	201 ± 64	174 ± 62
Protein (g/d)	81 ± 19	72 ± 16	82 ± 25	78 ± 29
Fat (g/d)	70 ± 20	67 ± 21	69 ± 20	66 ± 24
Dietary fiber (g/d)	25.3 ± 7.2	23.5 ± 8.4	23.8 ± 8.1	20.2 ± 6.4
Cholesterol (mg/d)	264 ± 105	229 ± 114	252 ± 126	221 ± 147
Energy distribution				
Carbohydrate (%)	44.2 ± 7.4	43.7 ± 8.0	44.3 ± 6.1	42.6 ± 6.8
Fat (%)	34.9 ± 6.9	35.9 ± 7.2	34.7 ± 6.5	35.7 ± 6.5
SAFA	12.5 ± 3.4	12.0 ± 3.3	12.6 ± 3.3	12.9 ± 3.6
MUFA	11.9 ± 3.0	12.8 ± 3.5	12.1 ± 3.1	12.7 ± 3.3
PUFA	6.0 ± 1.5	6.9 ± 2.3	6.0 ± 2.1	6.4 ± 1.9
Protein (%)	18.3 ± 3.3	17.7 ± 2.5	18.2 ± 3.1	19.1 ± 3.5

^1^Values are means ± SD. Not statistically tested. ^2^*Note*. Nutrients derived from test snack bars were not accounted in the nutrient calculation concerning background diet.

**Table 4:** Serum total and lipoprotein lipids^1^ during the study in placebo and stanol groups in all completed subjects (*n* = 70).

	Placebo group (*N* = 34)	Stanol group (*N* = 36)	Mean difference estimate^3^	*P*-value
Total cholesterol, mmol/L				
Screening	6.18 ± 0.55	6.16 ± 0.62		
Baseline	6.13 ± 0.53	6.05 ± 0.72		
4 weeks	6.22 ± 0.67	5.71 ± 0.63	−0.507	0.002^4^
Change from baseline^2^	0.09 ± 0.47	−0.34 ± 0.46	−0.449	<0.001^5^
% change from baseline^2^	1.6 ± 7.6	−5.2 ± 7.5	−6.806	<0.001^6^

LDL cholesterol, mmol/L				
Screening	4.26 ± 0.60	4.21 ± 0.61		
Baseline	4.17 ± 0.57	4.08 ± 0.71		
4 weeks	4.25 ± 0.67	3.79 ± 0.64	−0.459	0.004^4^
Change from baseline^2^	0.08 ± 0.45	−0.29 ± 0.41	−0.386	<0.001^5^
% change from baseline^2^	2.2 ± 10.7	−6.4 ± 10.3	−8.622	0.001^6^

HDL Cholesterol, mmol/L				
Screening	1.79 ± 0.44	1.82 ± 0.45		
Baseline	1.75 ± 0.39	1.76 ± 0.45		
4 weeks	1.67 ± 0.38	1.67 ± 0.41	0.002	NS^4^
Change from baseline^2^	−0.07 ± 0.18	−0.09 ± 0.25	−0.011	NS^5^
% change from baseline^2^	−3.8 ± 9.9	−4.0 ± 13.3	−0.112	NS^6^

Triglyceride, mmol/L				
Screening	1.18 ± 0.48	1.07 ± 0.43		
Baseline	1.17 ± 0.44	1.18 ± 0.53		
4 weeks	1.31 ± 0.58	1.20 ± 0.54	−0.104	NS^4^
Change from baseline^2^	0.14 ± 0.36	0.02 ± 0.35	−0.115	NS^5^
% change from baseline^2^	13.1 ± 30.5	4.1 ± 26.2	−9.023	NS^6^

Non-HDL cholesterol				
Screening	4.40 ± 0.63	4.34 ± 0.66		
Baseline	4.38 ± 0.59	4.29 ± 0.74		
4 weeks	4.55 ± 0.75	4.04 ± 0.69	−0.508	0.004^4^
Change from baseline^2^	0.17 ± 0.43	−0.25 ± 0.39	−0.426	<0.001^5^
% change from baseline^2^	3.8 ± 9.6	−5.4 ± 9.4	−9.156	<0.001^6^

^1^Values are means ± SD. To convert cholesterol and triglyceride values to mg/dL, multiply by 38.67 and 88.57 respectively; ^2^Absolute and percentage change based on individual data, (4 weeks-baseline)/baseline × 100; ^3^Mean Difference Estimate between Placebo and Stanol group in change or mean variable; ^4^*P* -value for significance of difference between Placebo and Stanol group tested with ANOVA model (equal to independent samples *t*- test); ^5^*P*-value for significance of difference between Placebo and Stanol group tested with ANCOVA model adjusted for baseline concentrations; ^6^*P*-value for significance of difference between Placebo and Stanol group tested with ANOVA model (equal to independent samples *t*- test).

## References

[1] Law M. (2000). Plant sterol and stanol margarines and health.

[2] Katan M. B., Grundy S. M., Jones P., Law M., Miettinen T., Paoletti R. (2003). Efficacy and safety of plant stanols and sterols in the management of blood cholesterol levels.

[3] Plat J., Mackay D., Baumgartner S. (2012). Progress and prospective of plant sterol and plant stanol research: report of the Maastricht meeting.

[4] Hallikainen M. A., Sarkkinen E. S., Gylling H., Erkkilä A. T., Uusitupa M. I. J. (2000). Comparison of the effects of plant sterol ester and plant stanol ester-enriched margarines in lowering serum cholesterol concentrations in hypercholesterolaemic subjects on a low-fat diet.

[5] Musa-Veloso K., Poon T. H., Elliot J. A., Chung C. (2011). A comparison of the LDL-cholesterol lowering efficacy of plant stanols and plant sterols over a continuous dose range: results of a meta-analysis of randomized, placebo-controlled trials.

[6] Ostlund R. E., McGill J. B., Zeng C.-M. (2002). Gastrointestinal absorption and plasma kinetics of soy Δ5-phytosterols and phytostanols in humans.

[7] Gylling H., Plat J., Turley S. (2014). Plant sterols and plant stanols in the management of dyslipidaemia and prevention of cardiovascular disease.

[8] Mackay D. S., Jones P. J. H., Myrie S. B., Plat J., Lütjohann D. (2014). Methodological considerations for the harmonization of non-cholesterol sterol bio-analysis.

[9] EFSA Panel on Dietetic Products and Nutrition and Allergies (2012). Scientific Opinion on the substantiation of a health claim related to 3 g/day plant sterols/stanols and lowering blood LDL-cholesterol and reduced risk of (coronary) heart disease pursuant to Article 19 of Regulation (EC) No 1924/2006.

[10] Mensink R. P., Ebbing S., Lindhout M., Plat J., van Heugten M. M. A. (2002). Effects of plant stanol esters supplied in low-fat yoghurt on serum lipids and lipoproteins, non-cholesterol sterols and fat soluble antioxidant concentrations.

[11] Miettinen T. A., Puska P., Gylling H., Vanhanen H., Vartiainen E. (1995). Reduction of serum-cholesterol with sitostanol-ester margarine in a mildly hypercholesterolemic population.

[12] Blair S. N., Capuzzi D. M., Gottlieb S. O., Nguyen T., Morgan J. M., Cater N. B. (2000). Incremental reduction of serum total cholesterol and low-density lipoprotein cholesterol with the addition of plant stanol ester-containing spread to statin therapy.

[13] Athyros V. G., Kakafika A. I., Papageorgiou A. A. (2011). Effect of a plant stanol ester-containing spread, placebo spread, or mediterranean diet on estimated cardiovascular risk and lipid, inflammatory and haemostatic factors.

[14] Hallikainen M. A., Uusitupa M. I. J. (1999). Effects of 2 low-fat stanol ester-containing margarines on serum cholesterol concentrations as part of a low-fat diet in hypercholesterolemic subjects.

[15] Gylling H., Radhakrishnan R., Miettinen T. A. (1997). Reduction of serum cholesterol in postmenopausal women with previous myocardial infarction and cholesterol malabsorption induced by dietary sitostanol ester margarine: women and dietary sitostanol.

[16] Plat J., van Onselen E. N. M., van Heugten M. M. A., Mensink R. P. (2000). Effects on serum lipids, lipoproteins and fat soluble antioxidant concentrations of consumption frequency of margarines and shortenings enriched with plant stanol esters.

[17] Plat J., Baumgartner S., Mensink R. P. (2015). Mechanisms underlying the health benefits of plant sterol and stanol ester consumption.

[18] Kriengsinyos W., Wangtong A., Komindr S. (2015). Serum cholesterol reduction efficacy of biscuits with added plant stanol ester.

[19] Clifton P. (2015). Influence of food matrix on sterol and stanol activity.

[20] Helldán A., Raulio S., Kosola M., Tapanainen H., Ovaskainen M.-L., Virtanen S. Finravinto 2012 -tutkimus – The National FINDIET 2012 Survey. ISBN 978-952-245-950-3 (painettu); 978-952-245-951-0 (web) THL. Raportti 16/2013, 187 s. Helsinki 2013.

[21] Doornbos A. M. E., Meynen E. M., Duchateau G. S. M. J. E., van der Knaap H. C. M., Trautwein E. A. (2006). Intake occasion affects the serum cholesterol lowering of a plant sterol-enriched single-dose yoghurt drink in mildly hypercholesterolaemic subjects.

[22] Keszthelyi D., Knol D., Troost F. J., van Avesaat M., Foltz M., Masclee A. A. M. (2013). Time of ingestion relative to meal intake determines gastrointestinal responses to a plant sterol-containing yoghurt drink.

[23] Polagruto J. A., Wang-Polagruto J. F., Braun M. M., Lee L., Kwik-Uribe C., Keen C. L. (2006). Cocoa flavanol-enriched snack bars containing phytosterols effectively lower total and low-density lipoprotein cholesterol levels.

[24] Friedewald W. T., Levy R. I., Fredrickson D. S. (1972). Estimation of the concentration of low-density lipoprotein cholesterol in plasma, without use of the preparative ultracentrifuge.

[25] Clifton P. M., Noakes M., Sullivan D. (2004). Cholesterol-lowering effects of plant sterol esters differ in milk, yoghurt, bread and cereal.

[26] Yoshida M., Vanstone C. A., Parsons W. D., Zawistowski J., Jones P. J. H. (2006). Effect of plant sterols and glucomannan on lipids in individuals with and without type II diabetes.

[27] Söderholm P. P., Alfthan G., Koskela A. H., Adlercreutz H., Tikkanen M. J. (2012). The effect of high-fiber rye bread enriched with nonesterified plant sterols on major serum lipids and apolipoproteins in normocholesterolemic individuals.

[28] Gylling H., Simonen P. (2015). Phytosterols, phytostanols, and lipoprotein metabolism.

[29] von Bergmann K., Sudhop T., Lütjohann D. (2005). Cholesterol and plant sterol absorption: recent insights.

